# Adenocarcinoma Arising in Adenomyosis: A Narrative Review of Disease Concept, Molecular Pathogenesis, and Clinical Challenges

**DOI:** 10.7759/cureus.104162

**Published:** 2026-02-24

**Authors:** Hiroki Egashira, Hiroaki Ishida, Akiko Takashima

**Affiliations:** 1 Obstetrics and Gynecology, Toho University Sakura Medical Center, Sakura, JPN

**Keywords:** adenocarcinoma arising in adenomyosis (aaia), adenomyosis, adenomyosis-associated endometrial carcinoma (aaec), conventional endometrial carcinoma with coexisting adenomyosis (ecwa), endometrial carcinoma, kras mutation

## Abstract

Adenomyosis has long been regarded as a benign, estrogen-dependent uterine disorder. Accumulating pathological and molecular evidence now supports its role as a potential precursor lesion for endometrial carcinoma. Adenocarcinoma arising in adenomyosis (AAIA), in particular, represents a rare but clinically significant entity characterized by malignant transformation within adenomyotic lesions of the myometrium. Adenomyotic lesions exhibit local estrogen excess, progesterone resistance, and a chronically inflamed microenvironment. Molecular studies indicate that adenomyosis may constitute a clonal disease harboring somatic driver mutations shared with endometrioid carcinoma, including KRAS mutations and alterations in the PI3K-AKT-mTOR signaling pathway. These observations support a multistep carcinogenesis model in which endometrial glands within adenomyosis accumulate genetic and epigenetic alterations, progress through atypical hyperplasia-like changes, and ultimately develop into invasive carcinoma. Adenomyosis-associated endometrial carcinoma encompasses two distinct pathological conditions: true carcinoma arising within adenomyosis (AAIA) and conventional endometrial carcinoma coexisting with adenomyosis. Accurate differentiation between these entities is essential, as AAIA often lacks an identifiable primary endometrial lesion and may therefore escape detection by conventional endometrial cytology or biopsy. In such cases, MRI, complemented by molecular pathological evaluation, plays a central diagnostic role. Management of AAIA generally follows established treatment strategies for endometrial carcinoma. However, advances in molecular classification, particularly those derived from The Cancer Genome Atlas, emphasize the importance of molecular subtype-based prognostic stratification and individualized therapeutic decision-making. Accordingly, this narrative review synthesizes current evidence on the disease concept, pathophysiology, molecular alterations, clinical characteristics, diagnostic challenges, treatment strategies, and future directions of endometrial carcinoma arising in adenomyosis.

## Introduction and background

Adenomyosis is a benign uterine disorder characterized by ectopic endometrial glands and stroma within the myometrium, accompanied by reactive smooth muscle hyperplasia. Clinically, it presents with dysmenorrhea, menorrhagia, and chronic pelvic pain, and diagnosis relies primarily on imaging findings, particularly MRI [1]. By contrast, endometrial carcinoma is the most common gynecologic malignancy among postmenopausal women, with abnormal uterine bleeding as the predominant presenting symptom. In the context of increasing obesity and metabolic disorders, the global incidence and disease burden of endometrial carcinoma have steadily increased [2].

Historically, adenomyosis and endometrial carcinoma were considered unrelated diseases. This assumption reflected differences in age at onset, distinct biological behaviors, and the rarity of carcinomas arising directly from adenomyosis. Furthermore, distinguishing carcinomas originating within adenomyotic lesions from conventional endometrial carcinomas secondarily invading adenomyosis has long posed a diagnostic challenge [3].

However, recent advances in pathological assessment and molecular analyses demonstrated that malignant transformation can occur within adenomyotic lesions [4]. These findings suggest that, in selected cases, adenomyosis may function as a precursor lesion for endometrial carcinoma. Although cases confined to adenomyosis often show relatively favorable outcomes, others, depending on histological subtype and molecular risk profile, experience recurrence or disease progression, underscoring the importance of early diagnosis. Clinically, symptoms attributable to adenomyosis may obscure malignant changes, and because tumors often develop within the myometrium, endometrial cytology or biopsy frequently yields negative results [4]. In this review, we focus on adenocarcinoma arising in adenomyosis (AAIA) and discuss its disease concept, pathophysiology, molecular alterations, clinical features, diagnostic challenges, therapeutic strategies, and future perspectives.

This article presents a narrative review integrating recent molecular, pathological, and clinical evidence to clarify the disease concept of AAIA and to provide practical insights for its recognition and management in daily clinical practice.

## Review

Methods

This narrative review summarizes the current evidence on endometrial carcinoma arising in adenomyosis. A comprehensive literature search was conducted using PubMed and included articles published up to December 2025. The search terms comprised “adenomyosis”, “adenocarcinoma arising in adenomyosis”, “adenomyosis-associated endometrial carcinoma”, and related keywords. English-language case reports, case series, molecular and pathological studies, systematic reviews, and relevant narrative reviews were included. Articles were selected based on relevance to disease mechanisms, diagnostic approaches, and clinical management.

Pathophysiology of adenomyosis and endometrial carcinogenesis

Pathological Features and Hormone Dependence

Adenomyosis is characterized by ectopic endometrial glands and stroma within the myometrium, accompanied by hyperplasia and hypertrophy of the surrounding smooth muscle. These ectopic endometrial glands often retain cyclical hormonal responsiveness similar to that of the eutopic endometrium; therefore, adenomyosis is considered an estrogen-dependent disease [5].

Adenomyotic lesions exhibit increased estrogen receptor expression and reduced progesterone responsiveness, a phenomenon termed progesterone resistance [6]. In particular, reduced expression of the progesterone receptor B isoform impairs progesterone-mediated antiproliferative effects [7]. In addition, adenomyotic tissues demonstrate aberrant aromatase expression, reduced estradiol inactivation, and enhanced local conversion from circulating estrogen precursors, thereby sustaining a locally hyperestrogenic state that is largely independent of systemic hormonal conditions [8,9].

Molecular Alterations in Adenomyosis

Recent molecular studies suggest that adenomyosis is not merely reactive or hyperplastic but may represent a clonal disease harboring somatic genetic mutations. Among these, KRAS mutations and activation of the phosphatidylinositol 3-kinase-AKT-mammalian target of rapamycin (PI3K-AKT-mTOR) signaling pathway are reported most frequently [10,11].

KRAS mutations, particularly hotspot mutations at codon 12, occur in a substantial proportion of endometrial glands within adenomyotic lesions and correspond to established driver mutations observed in malignant tumors [12]. These mutations are identified in approximately 20-40% of adenomyotic endometrial glands, a frequency significantly higher than that observed in normal endometrium, supporting clonal expansion within adenomyotic lesions [10,13].

Constitutively activated KRAS drives persistent mitogen-activated protein kinase/extracellular signal-regulated kinase pathway activation, promoting cell proliferation, resistance to apoptosis, and enhanced invasive capacity. These effects likely facilitate the survival and expansion of endometrial epithelial cells within the ectopic myometrial environment [14].

Additionally, molecular alterations involving the PI3K-AKT-mTOR pathway, including PIK3CA mutations and phosphatase and tensin homolog abnormalities, are also detected in adenomyotic lesions. These alterations overlap with those observed in endometrioid endometrial carcinoma, providing further evidence for a biological continuum between adenomyosis and malignant transformation [10,11].

Chronic Inflammation and the Repetitive Tissue Injury and Repair Hypothesis

Adenomyosis is associated with chronic inflammation driven by repetitive tissue injury and repair. Recurrent microhemorrhage, iron deposition, oxidative stress, and sustained inflammatory cytokine release contribute to a tumor-promoting microenvironment [15,16]. Activation of inflammatory signaling pathways, including nuclear factor kappa B, cyclooxygenase-2 (COX-2)/prostaglandin E2 (PGE2), and IL-6/signal transducer and activator of transcription 3, induces sustained cell proliferation, resistance to apoptosis, angiogenesis, and genomic instability, thereby facilitating carcinogenesis [17,18].

PGE2 further enhances estrogen production by inducing aromatase expression, whereas estrogen upregulates COX-2 expression and inflammatory cytokine production. This self-amplifying COX-2-PGE2-aromatase loop sustains chronic estrogen excess and inflammation within adenomyotic lesions, contributing to lesion growth, pain, and therapeutic resistance [19].

Adenomyosis as a Precursor Lesion for Carcinogenesis: An Integrated Model

**Figure 1:**
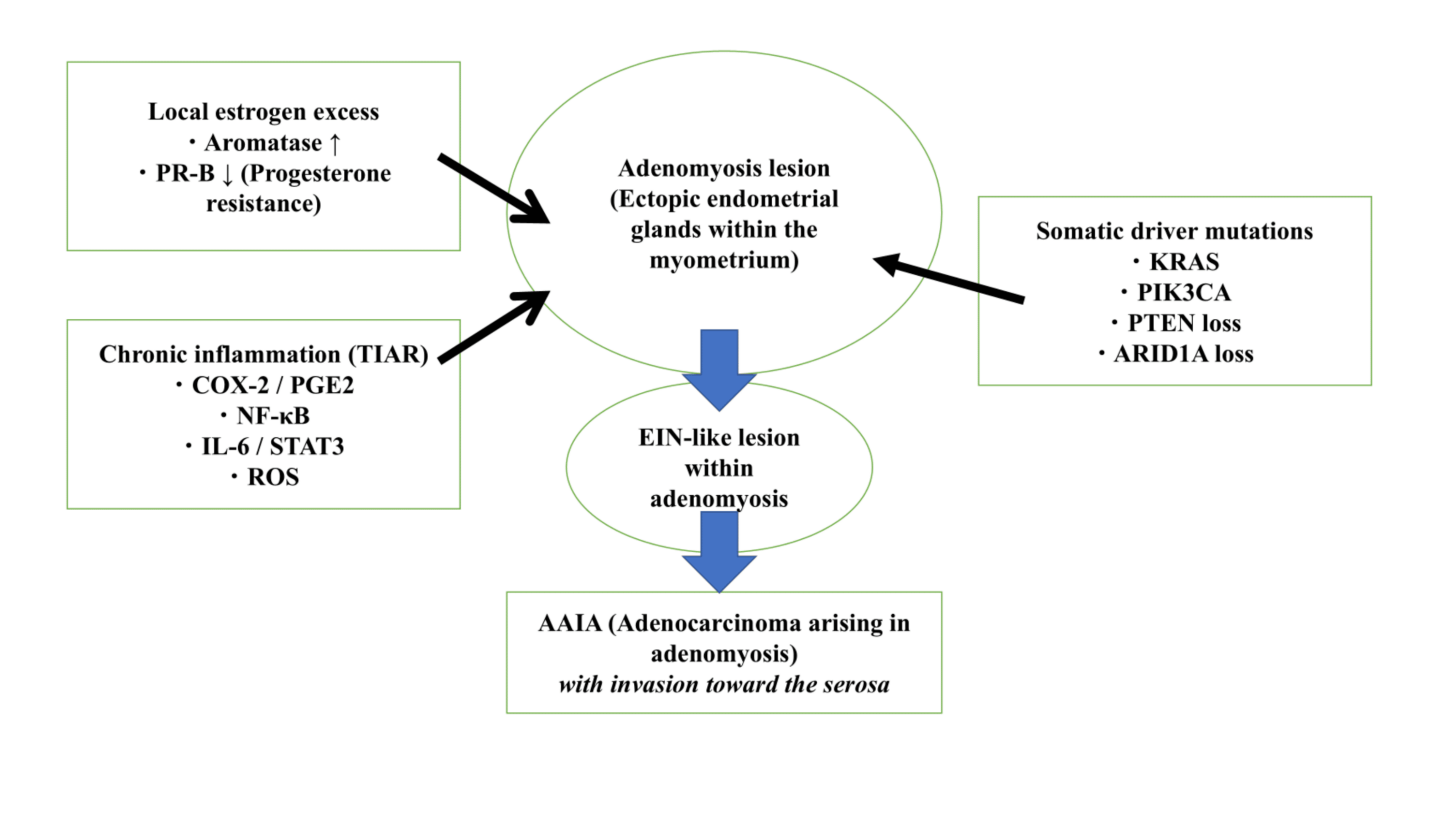
Integrated multistep carcinogenesis model of AAIA Adenomyotic lesions provide a tumor-promoting niche characterized by local estrogen excess and progesterone resistance, a chronic inflammatory microenvironment based on TIAR, and accumulation of somatic driver mutations, including KRAS, PIK3CA, PTEN, and ARID1A. These factors cooperatively promote clonal expansion of ectopic basal endometrial glands, development of EIN-like lesions within adenomyosis, and subsequent progression to invasive carcinoma (AAIA) extending toward the serosal surface. AAIA, adenocarcinoma arising in adenomyosis; EIN, endometrial intraepithelial neoplasia; TIAR, tissue injury and repair

When pathological features, hormone dependence, molecular alterations, and the chronic inflammatory microenvironment are considered together, adenomyosis can be positioned as a precursor lesion with a biological background conducive to carcinogenesis [5-19]. These integrated mechanisms are summarized in Figure 1.

Notably, several studies have reported cases in which adenomyotic lesions and associated endometrial carcinomas shared identical KRAS or PIK3CA mutations, providing molecular pathological evidence of origin from a common clonal ancestor [10].

Adenomyosis-associated endometrial carcinoma (AAEC)

Disease Concept and Classification

AAEC is a collective term for endometrial carcinomas arising in the background of adenomyosis and comprises two distinct pathological entities. These include true AAIA, which originates directly within adenomyotic lesions, and conventional endometrial carcinoma with coexisting adenomyosis (ECWA). AAIA is defined by the absence of an obvious primary tumor in the endometrial cavity and by malignant transformation within adenomyotic foci [20].

AAIA is extremely rare, accounting for less than 0.3-1% of all endometrial carcinomas, and most reported cases consist of isolated case reports or small case series [4,20]. By contrast, ECWA is relatively common, with adenomyosis coexisting in approximately 20-40% of endometrial carcinoma cases and representing a frequent concomitant finding [21].

Pathological Diagnostic Criteria for Carcinoma Arising in Adenomyosis

The diagnosis of AAIA has traditionally relied on the Sampson-Colman criteria, which require (1) absence of a primary tumor within the endometrial cavity; (2) demonstrable continuity between benign endometrial glands in adenomyosis and malignant epithelium; and (3) residual adenomyotic structures surrounding the tumor [4]. However, complete exclusion of an occult primary endometrial lesion remains challenging in routine clinical practice.

Recent studies identified shared somatic mutations between adenomyotic lesions and associated carcinomas, providing molecular evidence of continuity. These molecular pathological findings are increasingly recognized as valuable adjuncts to conventional morphological diagnosis [10].

Differential Diagnosis

Accurate differentiation between AAIA and conventional endometrial carcinoma secondarily invading adenomyosis is critical. The differential diagnosis also includes deep myometrial invasion by a microscopic endometrial carcinoma, uterine sarcoma, and carcinosarcoma. Establishing an accurate diagnosis often requires meticulous pathological evaluation, including serial sectioning, immunohistochemical analysis, and, in selected cases, molecular testing [10,22,23].

Classification of endometrial carcinoma and the position of AAIA

Endometrial carcinomas have traditionally been classified into estrogen-dependent type I and estrogen-independent type II tumors. Most reported cases of AAIA correspond to type I endometrioid carcinoma; however, high-grade histological subtypes, including clear cell and serous carcinomas, have also been reported [4,20].

According to the molecular classification proposed by The Cancer Genome Atlas, endometrial carcinoma is stratified into four molecular subgroups: POLE ultramutated, mismatch repair-deficient, p53-abnormal, and no specific molecular profile (NSMP). Limited data suggest that AAIA most commonly falls within the NSMP or mismatch repair-deficient categories, although poor-prognosis p53-abnormal tumors may also occur. Therefore, molecular classification remains essential for accurate risk stratification and therapeutic decision-making, regardless of tissue origin [24].

Mechanisms of carcinogenesis in adenomyosis

AAIA is best explained by a multistep carcinogenesis model. Endometrial glands originating from the basal layer migrate into the myometrium and acquire early driver mutations, including KRAS and PIK3CA. These altered cells persist within a locally hyperestrogenic and chronically inflamed microenvironment over prolonged periods. With the accumulation of additional genetic alterations, lesions progress through atypical hyperplasia-like changes and ultimately develop into invasive carcinoma [5-19].

Although the underlying molecular mechanisms largely overlap with those of conventional endometrioid endometrial carcinoma, the intramyometrial origin of AAIA accounts for its distinctive clinical presentation and diagnostic features.

Clinical characteristics

Most reported cases of AAIA occur in postmenopausal women in their 50s-60s, and the age at diagnosis is slightly higher than that of conventional endometrioid endometrial carcinoma [4,20]. Unlike typical endometrial carcinoma, abnormal uterine bleeding is frequently absent or minimal [1,3]. Instead, nonspecific symptoms such as pelvic pain or uterine enlargement often predominate [4]. These symptoms are commonly misattributed to the progression of benign adenomyosis, potentially delaying diagnosis.

MRI represents the most important diagnostic modality. On T2-weighted images, nodular or irregular hyperintense areas may appear within the characteristic hypointense regions of adenomyosis. Findings suggestive of malignancy include high signal intensity on diffusion-weighted imaging, low apparent diffusion coefficient values, and heterogeneous or early enhancement on contrast-enhanced MRI [25]. When tumors extend from the deep myometrium toward the serosal surface, locally advanced disease may be present despite minimal endometrial thickening, highlighting the importance of careful evaluation of intramyometrial lesions. ¹⁸F-fluorodeoxyglucose PET/CT may be used adjunctively to assess lymph node involvement and distant metastases [25].

Accordingly, the clinical characteristics of AAIA differ from those of conventional endometrial carcinoma (Table 1).

**Table 1:** Comparison between AAIA and conventional endometrial carcinoma The summarized information is based on prior studies [1,3,4,20,21,25]. AAIA, adenocarcinoma arising in adenomyosis; ADC, apparent diffusion coefficient; DWI, diffusion-weighted imaging; EIN, endometrial intraepithelial neoplasia; FDG, fluorodeoxyglucose; T2WI, T2-weighted imaging

Item	AAIA	Conventional endometrial carcinoma
Site of origin	Adenomyotic lesions located within the myometrium	Endometrial surface epithelium
Primary tumor location	Deep myometrium (ectopic endometrial glands within adenomyosis)	Endometrial lining
Endometrial involvement	Primary endometrial lesion absent or minimal; no continuous spread from the endometrium	Frequently associated with atypical endometrial hyperplasia/EIN or an obvious primary lesion
Initial symptoms	Lower abdominal pain, pelvic pressure, and uterine enlargement; abnormal uterine bleeding may be absent	Postmenopausal abnormal uterine bleeding is typical
Endometrial cytology/biopsy	May be negative due to lack of endometrial exposure	Often useful for diagnosis
Key imaging findings	MRI shows nodular lesions within low-signal adenomyotic areas on T2-weighted images, high signal intensity on DWI, low ADC values, and heterogeneous enhancement	Endometrial thickening, intracavitary mass, and assessment of myometrial invasion depth
Pattern of tumor spread	May progress from the deep myometrium toward the serosal surface, posing a risk of disease underestimation	Typically invades from the endometrial surface into the myometrium
Histopathological continuity	Direct transition from adenomyotic endometrial glands to atypical epithelium and invasive carcinoma	Continuous invasion from the endometrial epithelium
Molecular characteristics	Shared somatic mutations between adenomyotic lesions and carcinoma, including KRAS, PIK3CA, and PTEN, in some cases	Typical mutation spectrum of endometrial carcinoma derived from endometrial epithelium
Diagnostic criteria	Sampson-Colman criteria plus demonstration of molecular-pathological continuity	Standard diagnostic algorithm for endometrial carcinoma
Prognosis and recurrence pattern	Prognosis depends on molecular subtype; relatively higher incidence of peritoneal and lymph node recurrence has been reported	Prognosis depends on stage and molecular subtype; vaginal cuff recurrence is more common

Treatment and prognosis

Because AAIA is rare, disease-specific treatment guidelines have not been established. Management generally follows standard treatment principles for endometrial carcinoma, with surgery as the primary therapeutic approach. Indications for adjuvant treatment are based on disease stage, histological subtype, and molecular risk factors. Prognosis appears to depend more strongly on histological grade and molecular subtype than on tissue origin. In particular, high-grade histological subtypes and p53-abnormal tumors are associated with poor outcomes [4].

Future perspectives

Future challenges include standardization of diagnostic criteria incorporating molecular evidence, improvement of preoperative diagnostic accuracy, and clarification of molecular profiles through large-scale, multicenter studies to establish optimal treatment strategies. Advances in imaging, genomic analysis, and precision medicine are expected to improve outcomes while avoiding overtreatment.

## Conclusions

AAIA is a rare but clinically significant entity that supports the concept of adenomyosis as a potential precursor lesion for endometrial carcinoma. Owing to its intramyometrial origin and the frequent absence of an obvious endometrial lesion, AAIA may evade detection by conventional endometrial cytology or biopsy, underscoring the importance of heightened clinical awareness and careful radiologic assessment.

Accumulating evidence suggests that adenomyosis represents a biologically active condition characterized by local estrogen excess, chronic inflammation, and recurrent somatic driver mutations, thereby providing a permissive microenvironment for multistep carcinogenesis. An integrated diagnostic approach combining imaging, histopathological evaluation, and molecular profiling is therefore essential for accurate diagnosis, prognostic stratification, and individualized management. Further multicenter studies incorporating molecular classification are warranted to refine diagnostic criteria and establish optimal treatment strategies for this rare but clinically relevant disease entity.
